# Learning and assessment strategies to develop specific and transversal competencies for a humanized medical education

**DOI:** 10.3389/fphys.2023.1212031

**Published:** 2023-07-10

**Authors:** Antonio S. Tutor, Esther Escudero, María del Nogal Ávila, Juan Francisco Aranda, Hortensia Torres, Josué G. Yague, María José Borrego, Úrsula Muñoz, María C. Sádaba, Isabel Sánchez-Vera

**Affiliations:** Departamento de Ciencias Médicas Básicas, Facultad de Medicina, Universidad San Pablo-CEU, CEU Universities, Madrid, Spain

**Keywords:** competency-based learning, humanization, learning by doing, doctor-patient communication, role playing, rubric, student experiences, transversal and specific competencies

## Abstract

**Introduction:** Medical education should promote the development of skills and abilities that can be applied to real-world work performance. The aim of this study is to evaluate technical and methodological knowledge, as well as physician-patient communication skills, as one of the most important transversal competencies that a good physician should acquire; all this in a reliable, accurate and objective way.

**Methods:** We present a rubric specifically designed and implemented for the evaluation of specific and transversal competencies in the physiology practical sessions, during the second year of the medical degree. The assessment consists in two evaluation tests: 1) a theoretical test that consists of multiple-choice questions. Students must demonstrate that they have acquired adequate theoretical knowledge (specific competency “to know”); 2) a practical test, in which students are evaluated by the rubric through the simulation of a medical consultation. Thus, demonstrating their ability to execute/apply what they have learned in class (specific competency “to know how to do”). They are also evaluated on the transversal competencies that we call “communication with the patient” (transversal competency “to know how to be there”) and “dealing with the patient” (transversal competency “to know how to be”).

**Results:** We evaluated whether there were differences in the grades obtained by students when the transversal competencies were not assessed (academic years 2017-2018 and 2018-2019; n = 289), and when the transversal competencies were assessed by applying the rubric in the academic years 2019-2020, 2021-2022, and 2022-2023 (n = 526). Furthermore, we present a student perception that supports the use of clinical simulation and our rubric as a good method within the competency learning process.

**Discussion:** The acquisition of these competencies, starting from the first courses of undergraduate education, helps to raise the students’ awareness in the development of a more humanized medicine, allowing a better response to the patients’ needs. Our rubric, which clearly indicate the performance criteria, have become an excellent method to carry out the assessment of competencies, both for students and teachers, since they allow to obtain clear evidence of the level of acquisition and application of knowledge.

## 1 Introduction

It has been known for quite some time that academic records (acquired knowledge) do not always provide sufficient information to reliably predict people’s suitability for different jobs or successful careers (Barrett and Depinet, 1991; González Lorente and Martínez Clares, 2015). In this sense, the European Higher Education Area (EHEA), in its Bologna Declaration, established, as one of its fundamental objectives, the transformation of a teaching-centered education into a learning-centered education, in which the student must acquire a more active role in the whole process (Hannay, 2010; Goodman et al., 2018). A learning-centered education involves the development in the acquisition of competencies by students, expanding, without excluding, the traditional content-based approach (Candela et al., 2006; Christianson et al., 2007; Trullàs et al., 2022). Thus, competency-based learning is based on students learning how to transfer knowledge to reality, so that they know how to use it safely and effectively (Chacko, 2014; Lee and Chiu, 2022). Thus, current *curricula* are designed so that students acquire various competencies before completing the corresponding undergraduate program.

Martínez-Clares and Echeverría Samanes. (2009) listed the 4 types of competencies, specific and transversal, that each student must acquire during his or her program to perform satisfactorily in his or her professional career (Table 1).

**TABLE 1:** Definition of specific and transversal competencies (adapted from Clares, 2009).

1.- Specific competencies	Description
1.1 Technical Competency: TO KNOW	“A body of specialized knowledge related to a particular occupational field, which permits the expert mastery of the content and tasks inherent in the job”
1.2 Methodological competency: TO KNOW HOW TO DO	“Applying knowledge to specific work situations, using the most appropriate procedures, solving problems autonomously and transferring the experience gained to new situations"

2.- Transversal competencies	Description
2.1 Participatory competency: TO KNOW HOW TO BE THERE	“A set of attitudes and interpersonal skills that allow the person to interact in their work environment and develop their profession”
2.2 Social competency: TO KNOW HOW TO BE	“Personal characteristics and attitudes towards oneself, towards others and towards one’s profession, which allow for the optimal performance of professional activities”

The specific competencies are related to the acquisition of knowledge that the student must achieve by taking a specific subject. These competencies provide them with the technical skills or abilities they need to know for their future professional development, acquiring both the appropriate theoretical knowledge, “to know”, and the ability to know how to apply and put it into practice, “to know how to do”. But, in addition, as important as the acquisition of technical and methodological knowledge, is the acquisition of the so-called transversal competencies (Sá and Serpa, 2018), which are those that enable them to work in a team, to be flexible and adapt to different situations, to be reflective, analytical, respectful, empathetic, supportive, and to develop a commitment to others and promote ethics and values aimed at the pursuit of the common good, in short, “to know how to be there” and “to know how to be”.

This common framework for all undergraduate programs is particularly necessary in the health sciences degrees and more specifically in medicine, where the acquisition of transversal competencies is key, since they are extremely important for their practice, and they will apply throughout their professional career. So much so that medical students are assessed for these competencies before graduation through the Objective and Structured Clinical Evaluation (OSCE), which aims to evaluate skills and aptitudes in specific clinical situations (Carraccio and Englander, 2000). The OSCE is currently implemented in all medical schools in Spain, although its teaching is limited to the last years of the program. We strongly believe that it is important that students work on and acquire these skills from the beginning of their academic career. First, because this competency-based approach, applied from the first years of study when basic subjects predominate, will help students to become aware of the importance of this knowledge in their training process (Finnerty et al., 2010). Secondly, because students must finish their studies having acquired other skills that will be useful for them in the development of their professional performance, and for this, they must not only have the necessary theoretical knowledge, but also train the doctor-patient relationship as soon as possible (Belasen, 2018; Vogel et al., 2018). Good communication with patients is essential in medicine, since the patient’s cooperation and thus the outcome of the treatment depends to a large extent on it (Haskard et al., 2008). However, in addition to being able to convey information truthfully, they must do so with empathy, respect, and cordiality (Bakić-Mirić, 2008). It is increasingly necessary to treat the patient as a human being and not as a disease. There is a growing interest in improving the humanistic aspects of healthcare, which requires collaboration in the training of future professionals (Thibault, 2019).

Therefore, the implementation of competency-based learning in medical education is necessary, but it is also a great challenge. It may seem obvious, but it is important to emphasize, that competency-based education must be well designed, planned, and regulated to be effective (Hawkins et al., 2015; Gruppen et al., 2016; Caverzagie et al., 2017). Establishing a competency-based *curriculum* involves not only revising the definition of objectives and content, but also developing new strategies and methodological tools that enable its implementation and evaluation (Nigaard et al., 2008). It has been proposed to use a type of teaching based on active methods. These include role-playing**,** problem-based learning, the case method, and gamification, among others, which allow the development of skills such as critical thinking, analytical, argumentative, and reflective skills, as well as the promotion of oral communication skills (Goodman et al., 2018; Torralba and Doo, 2020). Therefore, one of the challenges that teachers face in current curricula is how to assess the competencies that students are supposed to acquire, for which it is necessary to plan the assessment systems appropriately and that they are consistent with the objectives pursued (Holmboe et al., 2010; Lockyer et al., 2017). Assessment by means of a rubric is one of the valid tools for this process, since the use of specific descriptors makes it possible to define levels of acquisition and, in addition, makes students aware of how their knowledge develops and provides them with an integrated view of the attitudes and actions for improvement that they can apply to new situations in different environments (Boateng et al., 2009; Martiáñez-Ramírez et al., 2022).

For all the above reasons, the aim of this study is to design a competency-based learning plan and an evaluation system that will allow us to adequately measure the level of competency-based progress acquired during practical physiology classes. In this way, the following objectives will be achieved:

- Encourage medical students to acquire specific and transversal competencies through role-plays that reflect application of theoretical concepts in practice and work on the doctor-patient relationship.
- To design an effective rubric that, independent of the evaluating teacher and the clinical case evaluated, allows an unbiased evaluation method that accurately reflects the acquisition of both transversal and specific competencies by the students in medical practice.

## 2 Methods

### 2.1 Learning and assessment plan

This experience was developed in the practical classes of the Physiology I subject during the second year of the Medical Degree. This subject is compulsory and consists of 7.5 ECTS credits. The theoretical part of the course covers general physiological phenomena, skeletal muscle and blood physiology, and the physiology of the cardiovascular and respiratory systems are studied. The practical classes are directly related to the theoretical content and are developed in 3-h sessions for 7 days, according to the following program (Table 2). The students were informed about the program of the practical classes and the evaluation system.

**TABLE 2:** Physiology practical classes syllabus.

Practical lesson number	Content
1	Physiology of skeletal muscle
2	Physiology of blood: Hemogram. Characterization of the blood type
3	Physiology of blood: Globular resistance. Clotting tests
4	Physiology of the cardiovascular system: Pulse. Cardiac auscultation. Blood pressure. Blood pressure regulation
5	Physiology of the cardiovascular system: Electrocardiogram
6	Physiology of the respiratory system: Respiratory mechanics. Spirometry
7	Physiology of the respiratory system: Vitalography

Throughout the physiology practices, students learn to: 1) measure blood pressure and interpret the obtained measurements, 2) to auscultate and take the pulse, checking whether the results are within physiological parameters, 3) to perform an electrocardiogram and analyze basic data from it, 4) to decipher the main parameters of a complete blood count and perform a blood type determination test, and finally 5) to conduct a spirometry and describe the obtained results. The practical activities are reinforced by the content of the theoretical classes. Each practical session has a maximum of 14 students, lasts for 3 h, and is designed as follows:

- Introduction: The professor provides the necessary theoretical knowledge to review the concepts associated with the clinical test that will be performed, as well as the basic notions to carry out the clinical procedure.
- Learning by doing: Students perform the clinical test in pairs, alternating between the roles of doctor and patient. The importance of doctor-patient communication is always emphasized. The professor directly supervises and advises the students, correcting their procedural approach and their interaction in the different roles. It was suggested that during the practical sessions they should simulate clinical care with their colleagues and put transversal competencies into practice. Thus, they were encouraged to be polite and cordial with the patient, to show interest in the patient´s health and the reason for the consultation, to explain the tests to be performed and finally to explain the results obtained.
- Learning by thinking: Students record the obtained data in their practice notebook and answer a series of questions related to the performance of the clinical procedure and the interpretation of the results obtained.
- Feedback: The practice concludes with a joint session between the professor and the students, reviewing the steps of the clinical procedure and the interpretation of the results. Emphasis is placed on how to convey information to patients.

This protocol provides a structure for students to acquire practical skills in the clinical field while reinforcing the theoretical concepts learned in class. The practical learning approach in pairs and constant feedback from the professor promote the development of technical skills and the improvement of doctor-patient communication.

The assessment consists of performing two evaluation tests: a theoretical test and a practical test. The final practical qualification is obtained based on 75% of the score of the theoretical test and 25% of the score of the practical test. The grading scale was always from zero to ten. The theoretical test consists of multiple-choice questions and students must demonstrate that they have acquired adequate theoretical knowledge (specific competency “to know”). The second test is a practical one, through the simulation of a medical consultation in which students alternate the roles of doctor or patient, thus having to demonstrate their ability to execute/apply what they have learned in class (specific competency “to know how to do”). In this second test, the students are also evaluated on the transversal competencies that we call “communication with the patient” (transversal competency “to know how to be there”) and “dealing with the patient” (transversal competency “to know how to be”). For the evaluation of this second practical test, a rubric was designed to evaluate the students more objectively.

### 2.2 Assessment of competencies. Design of the rubric

**FIGURE 1:**
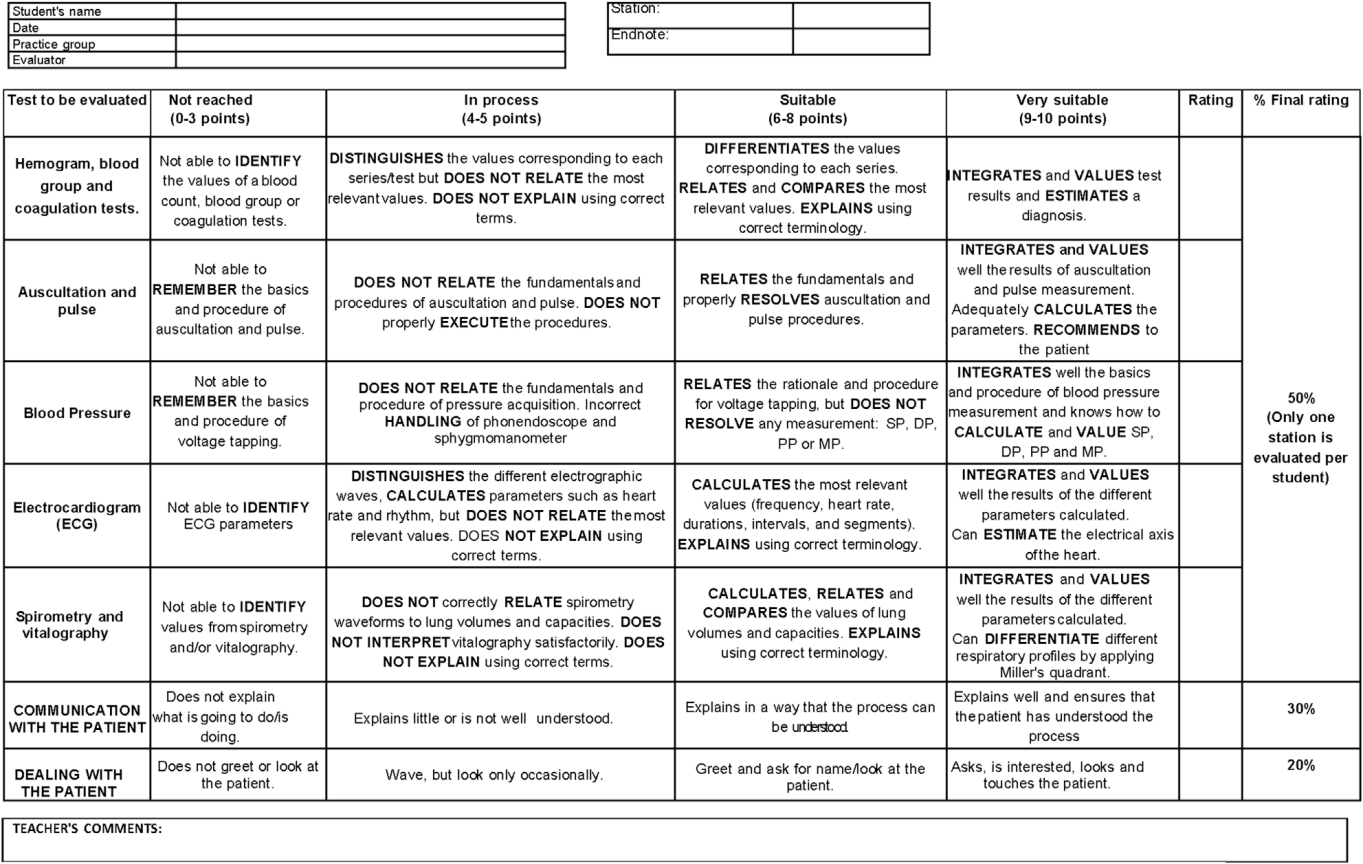
Competency assessment rubric. Tool that we have created to assess the theoretical knowledge, technical skills, and doctor-patient communication during Physiology practical sessions. Specific descriptors allow us to define levels of acquisition in each of the competencies we evaluate. *SP, Systolic Pressure; DP, Diastolic Pressure; PP, Pulse Pressure; MP, Medium Pressure.*

As an evaluation instrument for the practical test, a grading rubric (Figure 1) was designed to evaluate the theoretical knowledge and technical skills of the students, as well as the way they treated, behaved, and showed communication skills with a classmate who played the role of the patient. The rubric was designed according to Bloom’s scale (Bloom, 1956) and taken as a model some previous published rubrics (Moni et al., 2005; Ayhan and Türkyılmaz, 2015).

To assess the different competencies, the rubric was divided into several sections:

- First, the specific competency “to know-how to do” must be evaluated. The theoretical knowledge that the students had learned and their ability to put it into practice were evaluated, since they had to demonstrate that they could perform a procedure and know how to interpret the corresponding results. For this purpose, the evaluator, with the help of the rubric, selected and graded the students, according to their performance, in one of the following practical situations: determination of blood type, interpretation of a hemogram and clotting tests; cardiac auscultation and determination of heart rate; determination of blood pressure; interpretation of an electrocardiogram; interpretation of the results of spirometry and vitalography. The “to know how to do” competency assessment accounted for 50% of the final practical examination score.
- Secondly, during the practical examination, the evaluator had to evaluate the students’ ability to communicate with their partner, who played the role of the patient. This aspect therefore assessed the “to know how to be there” competency, as it considered the student’s ability to explain what test had been or would be performed, for what purpose, what the results obtained were and what the implications of these results were. This section was worth 30% of the final practical examination score.
- Finally, the last section of the rubric was used by the evaluator to assess the competency “to know how to be”, since during the practical classes the importance of this competency had been explained to the students and they had been encouraged to always be polite and cordial with the patient, to be empathetic, to be interested in the patient’s state of health and the reason for the consultation. This last part of the rubric accounted for the remaining 20% of the final grade.

All faculty involved in the teaching of the subject reviewed the content of the rubric and made suggestions about the dimensions and descriptors of the rubric. After incorporating the teachers’ comments, the rubric was considered valid for use. The assessment using the rubric was carried out during the academic years 2019-2020, 2021-2022 and 2022-2023.

Students are given advance information about the percentage of the grade that will be assigned to each part of the rubric. Although the weight of each competency in the final grade varies, students are made aware that they are all important to their overall education. They are explained that the primary objective of physiology practices is to learn how to perform and interpret various clinical tests and relate them to physiological processes, but they are also reminded of the significance we place on developing competencies that enable them to communicate and interact better with patients. Therefore, the “ to know how to do” competency carries more weight in the grading than the “to know how to be” and “to know-how to be there” competencies.”

### 2.3 Data collection

Competency assessment scores obtained by applying the rubric were collected from three different selected cohorts, these corresponded to students from the 2019-2020 academic year (n = 143), the 2021-2022 academic year (n = 179), and the 2022-2023 academic year (n = 204). Likewise, to evaluate whether there were differences in the grades obtained by the students when only specific competencies were evaluated, but not transversal competencies, the data were also collected for the two previous academic years, the 2017-2018 (n = 148) and the 2018-2019 (n = 141) academic years.

For the analysis of the data, the grades were grouped according to several variables such as: specific evaluations of the sections “to know” and “to know how to do”, “to know how to be there” and “to know how to be” sections, respectively, as well as the evaluating lecturer and the practical case to be evaluated.

### 2.4 Student perception. Opinion survey

**FIGURE 2:**
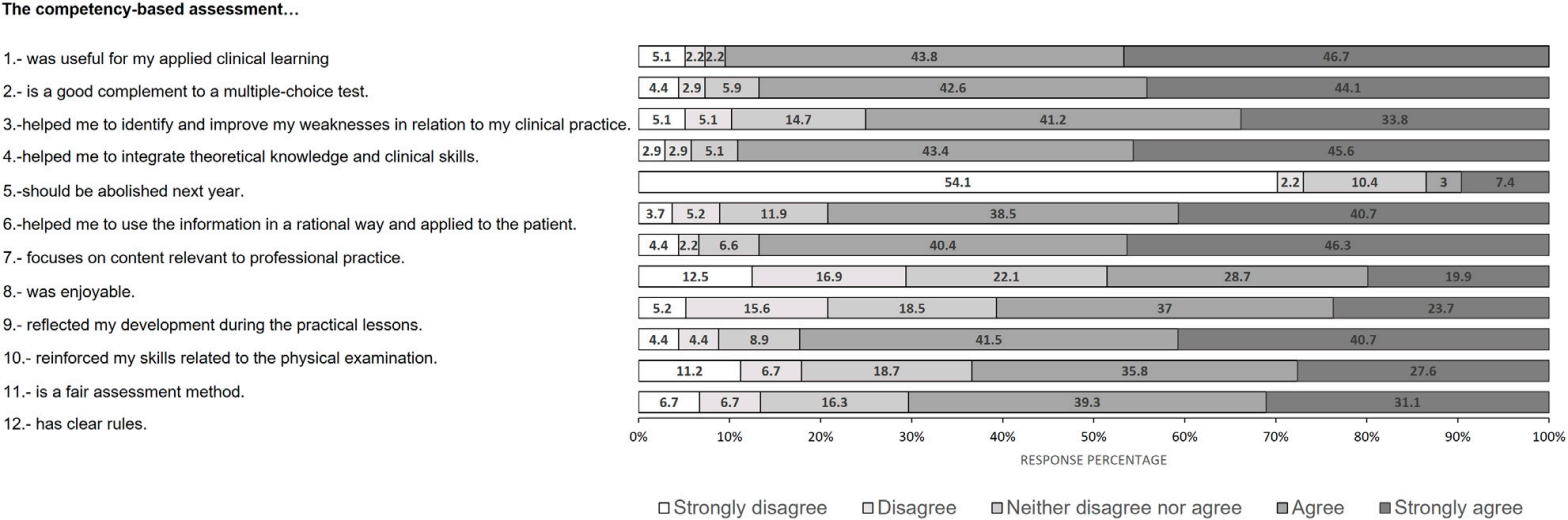
Results of the student opinion survey. Graph showing the percentages of the different response options (strongly disagree, disagree, neither agree nor disagree, agree, or strongly agree) to the different survey questions by the students.

The students’ perceptions of the usefulness and validity of the competency assessment were determined using an opinion survey. The survey consisted of 12 statements with which the students had to indicate their level of agreement (Likert scale with five levels: strongly disagree, disagree, neither agree nor disagree, agree, or strongly agree) and an open-ended question (Table 3; Figure 2). The survey was sent to all students in the 2022-2023 cohort. Of these, 137 students responded voluntarily and anonymously. For data analysis, the percentage of students who selected each level of the Likert scale was calculated.

**TABLE 3:** Questions for the perception survey on the assessment of competencies by students.

Question
1.- The competency assessment was useful for my applied clinical learning
2.- The competency-based assessment is a good complement to the evaluation method based on multiple-choice assessment
3.- The competency-based assessment helped me to identify and improve my weaknesses in relation to my clinical practice
4.- The competency-based assessment helps me to integrate theoretical knowledge and clinical skills
5.- The competency-based assessment should be abolished next year
6.- The competency-based assessment has helped me to use the information in a rational way and applied to the patient
7.- The competency-based assessment focuses on content relevant to professional practice
8.- I enjoyed doing the competency assessment
9.- The competency evaluation reflected my development during the practical lessons
10.- The competency-based assessment reinforces my skills related to the physical examination
11.- Competency-based assessment is a fair assessment method
12.- The assessment has clear rules
Open-ended question: Do you have anything to add regarding competency-based assessment?

This study was approved by the Ethics Committee of CEU San Pablo University (678/23/68). An informed consent was obtained from all students who wished to participate in the survey. Participation was voluntary and participants were assured of confidentiality.

### 2.5 Statistical analysis

Statistical analysis was preformed using the SPSS 27 program (SPSS Institute, France), with a *p*-value < 0.05 being considered statistically significant differences. The results were expressed as the mean ± standard deviation. The comparative study between the different courses analyzed was carried out using ANOVA for independent samples with subsequent Student-Newman-Keuls *post hoc* analysis for quantitative variables.

## 3 Results

### 3.1 Overall evaluation of the practical lessons of the subject: theoretical and practical knowledge

The average of the final practical qualification of the subject and the breakdown into the tests evaluating the theoretical and practical knowledge, since the academic years 2017-2018 to 2022-2023, are presented in Table 4.

**TABLE 4:** Mean scores obtained in the tests that evaluate the practical block of the subject: theoretical knowledge test, global practical test and final practical examination score in the different academic years. Data are presented as mean ± standard deviation. Data in the same column is compared using ANOVA with subsequent Student-Newman-Keuls *post hoc* analysis. (*) denotes statistically significant differences for the theorical knowledge test: * between 2017 and 2018 academic year with the others and ** between 2021 and 2022 academic year with the others; (#) denotes statistically significant differences for the global practical test between 2017-2018 and 2018-2019 academic years with the others, but there is no difference between each other. (§) denotes statistically significant differences for the final practical examination score: § between 2017 and 2018 academic year with the others and §§ between 2021 and 2022 academic year with the others. In all cases *p* < 0.05.

Academic year	Number of students	Theoretical knowledge test	Global practical test	Final practical examination score
2017-2018	148	8.09 ± 1.13^*^	8.70 ± 1.57^#^	8.24 ± 0.93^§^
2018-2019	141	7.27 ± 1.28	8.45 ± 1.58^#^	7.56 ± 0.85
2019-2020	143	7.21 ± 1.15	7.53 ± 1.58	7.29 ± 1.02
2021-2022	179	6.32 ± 1.61^**^	7.60 ± 1.75	6.64 ± 1.42^§§^
2022-2023	204	7.07 ± 1.37	7.91 ± 1.41	7.28 ± 1.32

As shown in Table 4, there is a variability in both the grades of the theoretical and practical tests and, therefore, in the final grade among the five academic courses analyzed.

The test that assesses theoretical knowledge and measures the level of mastery of the specific competency “to know” consists of multiple-choice questions drawn from a common bank of more than 100 questions of similar difficulty. This bank was developed by the teachers themselves and is accessible to all evaluators, but not to the students. The evaluators can choose the 30 questions they consider most appropriate, while maintaining a certain number of questions for each subject. Since this test is prepared every year using the same question bank, it is reasonable to assume that any statistically significant differences in scores between academic years are not due to differences in the actual difficulty of the test or to students’ prior knowledge of the test, but to the unique composition of students in each year considered.

However, when analyzing the results of the global practical knowledge test, we observed that there is a repeated pattern, being the results similar between the academic years 2017-2018 and 2018-2019, and between the academic years 2019-2020, 2021-2022 and 2022-2023. For the first two academic years analyzed, students obtained significantly higher scores (*p* < 0.001) than the three subsequent academic years. This fact is very relevant because in the first two courses analyzed, the rubric was not available as an evaluation method, but it was in the following three courses, when this assessment method was introduced. The fact that, until the 2018-2019 course, the grades in the practical test were higher may be due to the lack of objectivity that existed in the evaluation of the way the students performed the test. Since the rubric evaluation was established, we have observed that the grades are more unbiased and aligned to the development of the students in the subject.

### 3.2 Results of the evaluation of specific and transversal competencies in the global practical knowledge test

The use of the rubric to evaluate the doctor-patient simulation has allowed us to establish a global practical knowledge test that evaluates specific and transversal competencies in the same test. Thus, we can analyze both the skills developed by the students to perform a given clinical procedure and their ability to interact with the patient.

As is shown in Table 5, in the evaluation of the performance of the clinical procedure (specific competency “to know how to do”), there are no significant differences in the scores along the courses analyzed. In the analysis of the scores for the transversal competency “to know how to be” (“dealing with the patient”), there were also no statistically significant differences between the three last academic years, when this competency was evaluated using the rubric. Only in the analysis of the transversal competency “to know how to be there” (“communication with the patient”), we observe a statistically significant increase in the average grade obtained in the last course analyzed (2022-2023) when compared to previous academic years. This could be due to the consolidation of the rubric method over the years and the teacher’s greater insistence on the importance of developing this competency. This could be a determining factor for the students to become aware of its importance and to try to achieve it in the best possible way. In this sense, we can suggest that in recent years, we have noticed a significant increase in the interest and motivation of students to study careers related to health sciences. This increase can be attributed to the impact that the pandemic has had on society. The health crisis has highlighted the importance of having trained professionals who are committed to the health of people. In addition, the experience of the pandemic has raised a collective awareness of the need for more humane and empathetic treatment of patients. As a result, this increased motivation among students may have a positive impact on the quality of medical care in the future. Future healthcare professionals will be able to focus not only on the technical and scientific aspects, but also on the importance of establishing meaningful human connections with patients. It is crucial to encourage and support this increased interest in health science careers and to promote a comprehensive education that includes social and communication skills.

**TABLE 5:** Mean scores obtained on the rubric assessing specific and transversal competences in the different academic years. Data are presented as mean ± standard deviation. Data in the same column is compared using ANOVA with subsequent Student-Newman-Keuls *post hoc* analysis * denotes statistically significant differences between the 2022-2023 academic year and the others academic years analyzed for the competence “To know how to be there” (*p* < 0.05).

		Specific competency	Transversal competencies	
Academic year	Number of students	“To know how to do”	“To know how to be there” (communication with the patient)	“To know how to be” (dealing with the patient)
2019-2020	143	7.40 ± 2.04	7.53 ± 1.78	7.91 ± 1.76
2021-2022	179	7.54 ± 2.05	7.71 ± 1.84	7.59 ± 1.90
2022-2023	204	7.79 ± 1.66	8.12 ± 1.53^*^	7.91 ± 1.81
Total	526	7.60 ± 1.91	7.82 ± 1.72	7.80 ± 1.83

The average scores obtained by the students of the Faculty of Medicine over the last three academic years (n = 526) were 7.6 ± 1.91 for the competency “to know how to do”, 7.82 ± 1.72 for the competency “communication with the patient” and 7.8 ± 1.83 for the competency “dealing with the patient”. These passing grades indicate that the student has satisfactorily met the established evaluation criteria. This means that he/she has demonstrated an adequate level of knowledge and skills in relation to the objectives of the physiology practices. It is important to keep in mind that the rubric is not designed to produce high grades, but rather to fairly measure the student’s level of competency. The grades received indicate that the evaluation was balanced and provided an objective measure of the student’s level of competency.

### 3.3 Results of the evaluation of specific and transversal competencies according to the evaluator

One of the main concerns that we had was whether there was variability in the students’ grades in the practical test depending on the teacher who evaluated them. The differences in the grades obtained could be due either to the variability in the students’ learning or to the subjectivity of the evaluator. Therefore, one of the principal reasons to design the rubric was to have a simple grading system that would help different evaluators to objectively evaluate the students during their practical test.

Table 6 shows the results of the grades of specific and transversal competencies analyzed according to different evaluators in the academic years in which the rubric was used.

**TABLE 6:** Mean scores obtained by the rubric assessing specific and transversal competences according to the evaluators. Data are presented as mean ± standard deviation. Data in the same column is compared using ANOVA with subsequent Student-Newman-Keuls *post hoc* analysis *denotes statistically significant differences between evaluator 9 with the others evaluators for the competency “To know how to be there”. # denotes statistically significant differences between evaluators 7 and 10 for the competency “To know how to be there”. § denotes statistically significant differences between evaluators 7 and 8 for the competency “To know how to be”. In all cases *p* < 0.05.

Evaluator	Number of students	*“To know how to do”*	“To know how to be there” (communication with the patient)	“To know how to be” (dealing with the patient
Evaluator 1	33	6.62 ± 2.67	7.36 ± 2.1	7.44 ± 2.09
Evaluator 2	46	8.07 ± 1.64	7.82 ± 1.78	7.96 ± 1.57
Evaluator 3	182	7.49 ± 1.73	7.41 ± 1.71	7.33 ± 1.90
Evaluator 4	74	7.77 ± 2.17	7.93 ± 1.72	7.51 ± 2.06
Evaluator 5	22	7.90 ± 0.99	8.59 ± 1.20	8.22 ± 1.15
Evaluator 6	69	8.13 ± 1.97	8.66 ± 1.44	8.24 ± 1.66
Evaluator 7	10	7.10 ± 2.33	7.3 ± 1.56^#^	7.10 ± 1.79^§^
Evaluator 8	8	7.87 ± 1.72	8.37 ± 1.5	9.25 ± 0.71^§^
Evaluator 9	21	6.43 ± 1.74	6.05 ± 1.11^*^	8.76 ± 0.88
Evaluator 10	15	6.80 ± 1.94	8.86 ± 0.91^#^	8.86 ± 1.06
Evaluator 11	46	7.93 ± 1.48	8.45 ± 1.35	8.51 ± 1.65

It can be observed that there are no statistically significant differences in the grades of “to know how to do” competency of the students depending on the teacher who evaluated them.

However, in communication and patient deal, there are statistically significant differences in some mean scores among some evaluators. This could be due to a different acquisition of competencies by the students, since there is a positive correlation between the score of the practical case evaluated and the score obtained in communication with the patient (r = 0.666; *p* < 0.001; n = 526), as well as in dealing with the patient (r = 0.409; *p* < 0.001; n = 526). The results show a certain tendency among the different scores, such that students who have obtained better results in the tests that evaluate the specific competency “to know how to do” would obtain better results in the scores of the transversal competencies “to know how to be” and “know how to be there”, independently of the teacher who evaluated them. Even so, we cannot rule out the possibility of a student being able to satisfactorily perform a clinical procedure, but then not knowing how to communicate and deal with his or her patient adequately, or *vice versa*. Nor can we rule out that there is still some subjectivity on the part of certain evaluators who have not strictly adhered to the use of the rubric.

Considering all the above, and bearing in mind certain limitations, we dare to assert that the rubric designed is useful to minimize the variability that may exist in the objectivity of the evaluators and is an effective method that helps to evaluate both specific and transversal competencies.

### 3.4 Results of the evaluation of specific and transversal competencies according to the practical case evaluated

Table 7 shows the results obtained by analyzing the average grades for “to know how to do”, “to know how to be” and “to know how to be there” competencies according to the different practical cases evaluated, during the academic years in which the rubric was used (2019-2020, 2021-2022, and 2022-2023).

**TABLE 7:** Mean scores obtained by the rubric that evaluates specific and transversal competences according to the different practical cases. Data are presented as mean ± standard deviation. The number of students analyzed is shown in brackets. Data in the same column is compared using ANOVA with subsequent Student-Newman-Keuls *post hoc* analysis * denotes statistically significant differences between the 2019-2020 academic year and the others academic years analyzed for the competency “To know how to do” in the Blood pressure test (*p* < 0.05). ECG: Electrocardiogram. Different letters indicate statistically significant differences between the different courses analyzed (*p* < 0.001). ECG: Electrocardiogram.

Academic year	Hemogram and blood type	Auscultation and heart rate	Blood pressure	ECG	Spirometry and vitalography
“To know how to do”					
2019-2020	7.88 ± 1.88 (43)	7.31 ± 1.95 (24)	6.61 ± 2.1^*^ (28)	7.09 ± 2.31 (27)	7.97 ± 1.76 (21)
2021-2022	7.91 ± 1.8 (54)	6.7 ± 2.5 (32)	7.43 ± 1.53 (36)	7.53 ± 1.77 (27)	7.91 ± 2.55 (29)
2022-2023	7.59 ± 1.75 (70)	7.96 ± 1.49 (28)	8.01 ± 1.19 (36)	7.87 ± 1.83 (32)	7.79 ± 1.85 (38)
“To know how to be there”(Communication with the patient)					
2019-2020	7.65 ± 1.46 (43)	7.56 ± 1.42 (24)	7.07 ± 1.97 (28)	7.46 ± 2.16 (27)	7.97 ± 1.95 (21)
2021-2022	8.06 ± 1.85 (54)	7.20 ± 2.07 (32)	7.51 ± 1.68 (36)	7.37 ± 1.59 (27)	8.15 ± 1.84 (29)
2022-2023	7.73 ± 1.65 (70)	8.50 ± 1.31 (28)	8.27 ± 1.15 (36)	8.5 ± 1.26 (32)	8.11 ± 1.86 (38)
“To know how to be”(Dealing with the patient)					
2019-2020	7.66 ± 1.78 (43)	7.96 ± 1.75 (24)	7.73 ± 2.071 (28)	8.07 ± 1.54 (27)	7.78 ± 1.72 (21)
2021-2022	7.67 ± 1.82 (54)	7.45 ± 2.06 (32)	7.56 ± 1.79 (36)	7.48 ± 2.01 (27)	7.74 ± 2.03 (29)
2022-2023	7.63 ± 1.76 (70)	8.14 ± 1.63 (28)	8.18 ± 1.73 (36)	8.11 ± 1.86 (32)	7.85 ± 2.06 (38)

The data collected in the table shows that there are no statistically significant differences in the grades obtained for any of the competencies analyzed depending on the clinical case evaluated within the same course or between different courses. Exceptionally, we observe statistically significant differences only in the specific case of the competency “to know how to do” for blood pressure measurement in the 2019-2020 academic year. From these data we can conclude that in general there are no clinical cases that are more difficult than others.

### 3.5 Students’ perceptions of competency-based assessment

For a better analysis of the students’ responses (Figure 2), we have grouped the 12 statements of the survey into two different categories: those related to the way a competency was assessed (questions 2, 5, 7, 8, 9, 11, and 12) and those related to the usefulness of conducting this type of competency assessment (questions 1, 3, 4, 6, and 10).

Virtually all respondents agreed or strongly agreed with the statements that competency-based assessment was useful in their applied clinical learning (90.5%), has allowed them to integrate theoretical knowledge and clinical skills (89%), and strengthened their ability to perform a good physical examination of the patient (82.2%). In addition, a clear majority also agreed or strongly agreed with the statements that the competency-based assessment has helped them to use information in a rational and applied way (79.2%) and helps them to identify and improve their weaknesses related to clinical practice (75%).

Students agree or strongly agree that competency-based assessment is a good complement to the method based on multiple-choice assessment (86.7%) and that it focuses on content relevant to professional practice (86.7%). Most students agree or strongly agree that competency-based assessment is a fair way of evaluation (63.4%) and has clear rules (70.4%). On the other hand, very few (10.4%) agree or strongly agree that this type of competency-based assessment should be eliminated in the next course. In addition, almost half of the students (48.6%) claim to have enjoyed taking the test.

The survey included an open-ended question so that students could add any comments related to competency assessment. Some of the opinions expressed by the students were the following: *“I think it is a very good idea because we do not have any subject or any practice in which we can put our knowledge into action as doctors”*, *“Very useful for life and our future work”*, *“I think the oral practice exam is even more important than the theoretical one”, “It seems to me a very good way of evaluation because it makes you face a situation that we will have to face in the future and in which we apply the knowledge we have learned”.*

## 4 Discussion

The fact of accumulating knowledge does not necessarily imply being competent. Competence, in professional terms, is measured by the degree of resolution aptitude that people show in the development of their professional activity (Kukkonen et al., 2020). The relevance of all of this is visible in our daily work. It is common to associate good work performance with the ability to solve a problem; on the contrary, if they are not able to solve it, they can be labeled as incompetent and not knowing how to do their job well (García et al., 2020). In neither case are people’s intelligence, knowledge or preparation being judged, but rather how they managed to use it and apply all their training in the performance of their task. Education in competencies is education in knowledge, there is no other way to educate, but it implies a redefinition of curricula, orienting them towards the education of people in a broader sense (Timmerberg et al., 2022). In this way, we will guarantee our students a personal and intellectual development that will lead them to develop a complete education in line with the demands of the labor market (French et al., 2020). Comprehensive education is articulated based on conceptual, procedural, and attitudinal knowledge (Oandasan et al., 2020).

In the specific case of medical education, it is increasingly necessary that the efforts of the professors be focused on offering the students a complete, qualified, and competent training, where they are not only taught what they must know and be able to do, but also encouraged to develop more humanistic attitudes and to train the doctor-patient relationship (Weiss et al., 2019; Forsey et al., 2021). To be able to transmit information truthfully, but also with empathy, respect, and cordiality.

All of this leads us to promote strategies aimed at the acquisition of competencies by students, expanding but not excluding the traditional content-based path. However, although nobody doubts the above, it is necessary to emphasize the importance of a good development of the methods of learning these competencies, as well as the procedures to evaluate their level of acquisition. This requires evaluation systems that are in line with the objectives and that are known to the students, so that they know what is expected from them and are aware of their learning process, development, and personal and professional growth (Chimea, 2020).

Our main purpose in this work has been to instruct our students not only in the development of specific competencies, but also in the development of transversal competencies, specifically the doctor-patient relationship, from the beginning of their medical training and not to postpone it to higher courses (Daniel et al., 2021; Krishnasamy et al., 2022). To this end, we have used active methodologies based on simulation environments and role playing. In this way, students put into practice the knowledge acquired in different clinical situations and worked on the communication and treatment of the patient by informing him/her about the test procedure to be performed and/or transmitting the results of the test itself. The development of competency-based learning strategies must necessarily be accompanied by the design of specific and appropriate evaluation methods to assess the degree of acquisition of these competencies (Min Simpkins et al., 2019). The results further suggest that we have developed a rubric that allows us to appropriately measure the acquisition of the knowledge necessary to perform a given clinical test, as well as the acquisition of the ability to relate to the patient, regardless of the teacher performing the assessment or the clinical test in question.

The rubric provides students with measurable evidence of their learning process, achievement, and development of competencies, and is considered as a valid and a real alternative to traditional evaluation methods (Virk et al., 2020). Moreover, the use of the rubric increases transparency and reduces subjectivity in the evaluation process among the different evaluator, and therefore we can affirm that it makes the process fairer. Its inclusion in the evaluation of communication skills and patient care promotes the acquisition of essential competencies for the professional development of future physicians from the first courses.

Thus, we can consider competency assessment using the rubric, both teachers and students, as a valid complement to written exams (“know how”) and allows making students aware of the importance of striving to develop competencies (“to know how to do”, “to know how to be there” and “to know how to be”) to achieve a comprehensive training that allows them to develop as better professionals.

## Data Availability

The raw data supporting the conclusion of this article will be made available by the authors, without undue reservation.
